# Imaging study of midface growth with bone-borne trans-sutural distraction osteogenesis therapy in growing cleft lip and palate patients

**DOI:** 10.1038/s41598-018-37326-8

**Published:** 2019-01-29

**Authors:** Haizhou Tong, Tao Song, Xiaomei Sun, Ningbei Yin, Lei Liu, Xingang Wang, Zhenmin Zhao

**Affiliations:** 1 Department of Cleft Lip and Palate, Plastic Surgery Hospital, Chinese Academy of Medical Sciences and Peking Union Medical College, Beijing, China; 2 Department of Stomatology, Beijing Children’s Hospital, Capital Medical University, Beijing, China

## Abstract

Trans-sutural distraction osteogenesis (TSDO) promotes midface growth in growing cleft lip and palate (CLP) patients with midfacial hypoplasia. The superficial skeletal changes after therapy revealed rotation advancement of the midfacial skeleton associated with differential displacement in each segment, but reports rarely focus on the changes of internal structures, including circummaxillary sutures, the maxillary tuberosity and the maxillary sinus, which may play a crucial role during this process. This study evaluated the computed tomographic (CT) images of 26 growing CLP patients who received bone-borne TSDO therapy. The results revealed that the most prominent new bone formation occurred in the pterygomaxillary suture and pushed the P-point forward. The maxillary first molar exhibited significantly greater advancement compared with the P-point due to the growth of the maxillary tuberosity. The contribution ratio values of the advancement of the maxillary tuberosity and P-point to the maxillary first molar were 26% and 74%, respectively, in UCLP and 25% and 75%, respectively, in BCLP. Furthermore, the maxillary sinus volume was also significantly increased. In conclusion, midface growth with bone-borne TSDO therapy depends on both secondary displacement promoted by sutural bone formation mainly in the pterygomaxillary suture and primary displacement by growth of the maxillary tuberosity and maxillary sinus volume.

## Introduction

The normal growth and development of midfacial skeleton, which is defined as the nasomaxillary and zygomatic bones, depends on two fundamental mechanisms: secondary displacement promoted by the cranial base structure growth and primary displacement by the growth modelling of the skeleton itself. When the above-mentioned mechanisms are disturbed by genetic and environmental factors or acquired injuries during the early stage of life, it may lead to midfacial hypoplasia^1,2^.

Patients affected by cleft lip and palate (CLP) are most susceptible to develop into midfacial hypoplasia and typically present an asymmetrical or symmetrical concave facial profile associated with skeletal Class III malocclusion and narrow dental arch early in life^3,4^. This phenomenon is attributed to 2 aspects: intrinsic growth deficiency and iatrogenic factors caused by operation-based sequential therapy^5–7^. Their combined effects ultimately result in not only a retrusive midface position relative to the cranial base but also reduced maxillary size in three dimensions (3D)^8,9^. Therefore, the treatment targets of midfacial hypoplasia should include both the advancement of whole midfacial skeleton and the expansion of maxillary size to restore a harmonious facial appearance and occlusal relationship.

The common orthopaedic approach for mild to moderate midfacial hypoplasia in growing patients with CLP is maxillary protraction with tooth-borne or bone-borne anchorage, and the latter gradually occupies the dominant position by virtue of the advantage of decreasing unwanted dentoalveolar effects while increasing skeletal effects^10–13^. However, for a certain proportion of those with severe forms, maxillary protraction in either type cannot achieve adequate skeletal changes within less than 6 mm advancement of A-point according to current studies^14–16^. Recently, the technique of trans-sutural distraction osteogenesis (TSDO), which shares the same treatment principle with bone-anchored maxillary protraction using facemask, has just emerged to fill this gap^17,18^. Based on the rigid external distraction system with nickel-titanium shape memory alloy spring and bone-borne traction hooks anchored at the lateral nasal wall, this technique can acquire maximal advancement of the midfacial skeleton associated with maxillary expansion.

Numerous studies have conducted cephalometric 2-dimensional or 3-dimensional analysis to describe superficial skeletal changes after therapy^10–19^, but reports rarely focus on the changes of internal structures, which may play a crucial role in the growth and advancement of the midface. Therefore, the purpose of this study was to analyse the computed tomogram (CT) images of 26 growing CLP patients with midfacial hypoplasia undergoing bone-borne TSDO therapy and further elaborate the role of circummaxillary sutures, the maxillary tuberosity and the maxillary sinus in this process.

## Results

### Changes in circummaxillary sutures

**Figure 1:**
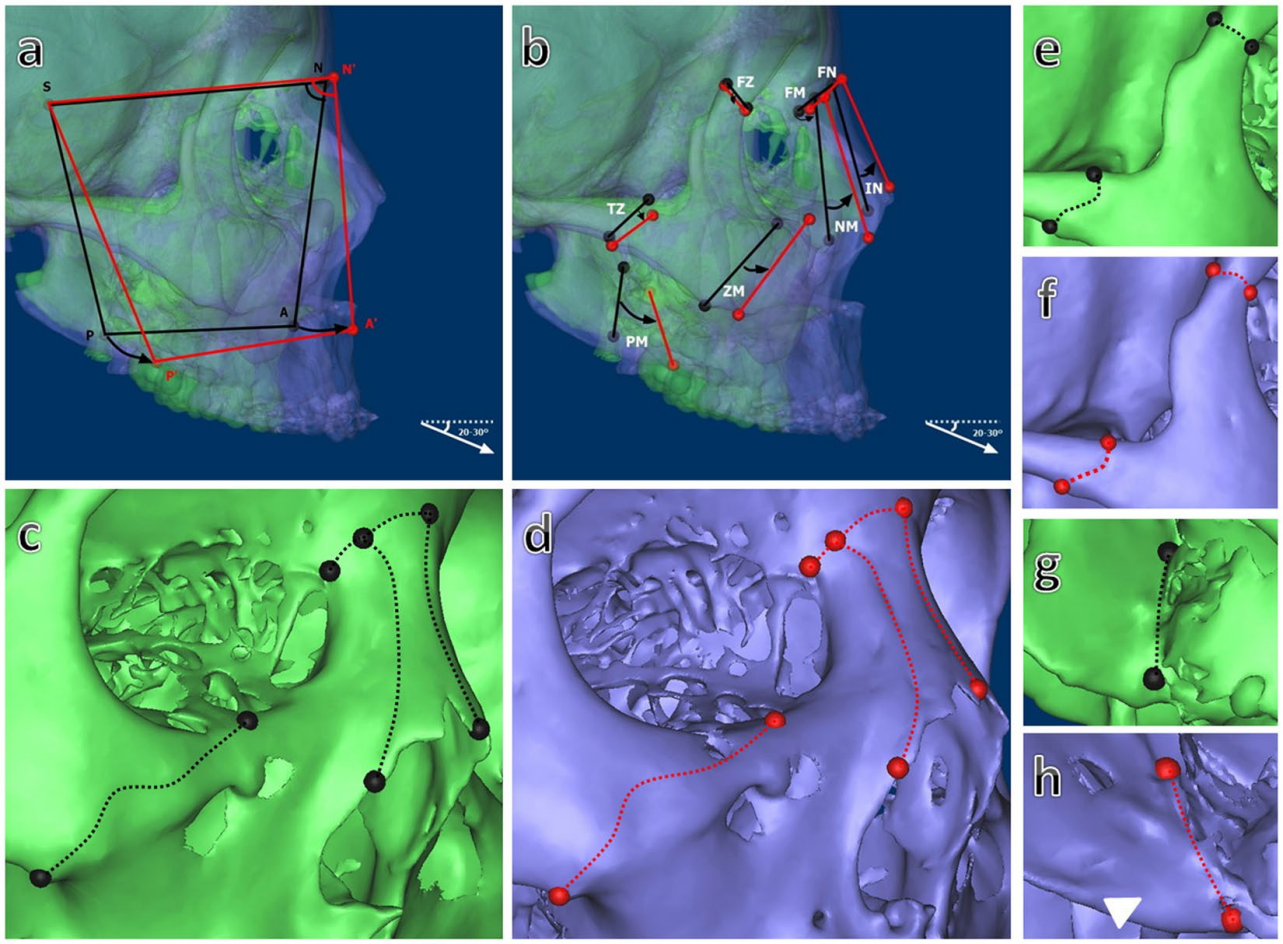
Changes in circummaxillary sutures between T0 (green) and T1 (purple). (**a**) The movement track of the P-point and A-point at the dentoalveolar level on the lateral view of semi-transparent superimposition images (T0: black; T1: red). (**b**) The movement track of endpoints of each circummaxillary suture on the lateral view of semi-transparent superimposition images (T0: black line; T1: red line). (**c**,**d**) The morphological changes of sutural contour in the region of the midfacial group (T0: black dash line; T1: red dash line). (**e**–**h**) The morphological changes of sutural contour in the region of the cranial group. White triangle marked the most prominent new bone formation in the pterygomaxillary suture. FN: frontonasal suture; FM: frontomaxillary suture; IN: internasal suture; NM: nasomaxillary suture; ZM: zygomaticomaxillary suture; FZ: zygomaticofrontal suture; TZ: zygomaticotemporal suture; PM: pterygomaxillary suture.

In the region of the cranial group between T0 and T1, the pterygomaxillary, zygomaticotemporal and zygomaticofrontal suture exhibited forward and downward displacement in descending order, whereas the frontomaxillary and frontonasal suture exhibited minor forward and upward displacement with 20–30° anteroinferior distraction force vector (Fig. 1b). In additon, the most prominent new bone formation was observed in the pterygomaxillary suture (Fig. 1e to h) and pushed the lower part of maxillary at the dentoalveolar level with an average forward displacement of 12.39 ± 4.71 mm at the P-point in UCLP (P < 0.001) and 16.27 ± 4.77 mm in BCLP (P < 0.001) and an average downward displacement of 5.50 ± 1.87 mm in UCLP (P < 0.001) and 7.38 ± 3.17 mm in BCLP (P < 0.05) (Fig. 1a; Table 1). In the region of the midfacial group, the internasal and nasomaxillary suture exhibited forward and upward displacement, whereas the zygomaticomaxillary suture exhibited major forward displacement (Fig. 1b). Similarly, the nasomaxillary and zygomaticomaxillary suture exhibited adaptive changes of sutural bone growth with tensile stresses to further push the middle-upper part of maxillary forward (Fig. 1c,d). The A-point exhibited an average forward displacement of 13.99 ± 4.90 mm in UCLP (P < 0.001) and 9.75 ± 4.56 mm in BCLP (P < 0.05) and minor upward displacement of 1.35 ± 2.90 mm in UCLP (P > 0.05) and 0.64 ± 4.03 mm in BCLP (P > 0.05) (Fig. 1a; Table 1).

**Table 1:** Comparison of the landmarks to 3D reference planes and measurements between T0 and T1.

Landmarks (mm)	UCLP (n = 20,T0-T1)			BCLP (n = 6,T0-T1)		
	CR	HR	MSR	CR	HR	MSR
A	13.99 ± 4.90*	−1.35 ± 2.90	0.52 ± 1.46	9.75 ± 4.56*	−0.64 ± 4.03	−1.03 ± 1.84
A_1_	16.26 ± 5.39*	0.74 ± 3.41	0.66 ± 2.24	15.05 ± 4.99*	0.60 ± 4.09	−0.69 ± 3.18
P_3M_	17.14 ± 6.00*	1.15 ± 2.63	−0.04 ± 1.35	21.15 ± 6.18*	3.64 ± 6.24	0.18 ± 0.71
P_6M_	16.71 ± 5.82*	3.66 ± 2.16*	0.05 ± 1.26	21.61 ± 5.95*	5.59 ± 2.79*	0.82 ± 0.96
P	12.39 ± 4.71*	5.50 ± 1.87*	0.29 ± 0.94	16.27 ± 4.77*	7.38 ± 3.17*	0.86 ± 0.93
Measurements	T0-T1			T0-T1		
P_3_W (mm)	3.03 ± 1.44*			5.67 ± 1.99*		
P_6_W (mm)	0.97 ± 3.40			2.76 ± 3.74		
A_1_-P_3M_⊥CR (mm)	−0.88 ± 1.22*			−6.10 ± 3.59*		
A_1_-P_6M_⊥CR (mm)	−0.45 ± 1.63			−6.57 ± 3.55*		
P_3M_-P_6M_⊥CR (mm)	0.43 ± 1.37			−0.46 ± 2.09		
P_6M_-P⊥CR (mm)	4.32 ± 1.90*			5.34 ± 1.27*		
P_6M_-P⊥CR/P_6M_⊥CR	0.26 ± 0.14			0.25 ± 0.02		
P⊥CR/P_6M_⊥CR	0.74 ± 0.14			0.75 ± 0.02		
V_MS_ (mm^3^)	1920.73 ± 1345.61*(left side) 2004.72 ± 1610.87*(right side)			3346.42 ± 1799.01*(left side) 3166.21 ± 2082.71*(right side)		

Data presented as the mean ± standard deviation. See Table 2 for the definitions of the landmarks, planes and measurements. *P < 0.05.

### Changes in the maxillary tuberosity and maxillary sinus

**Figure 2:**
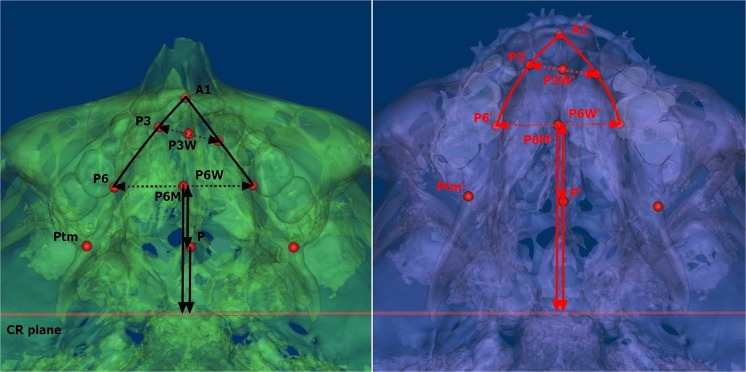
Changes in the measurements of P_3_W, P_6_W (dash line with arrows), P_6M_⊥CR, P⊥CR and P_6M_-P⊥CR (solid line with arrows perpendicular to CR plane) between T0(Left) and T1(Right) on antapical view of the semi-transparent images.

**Figure 3:**
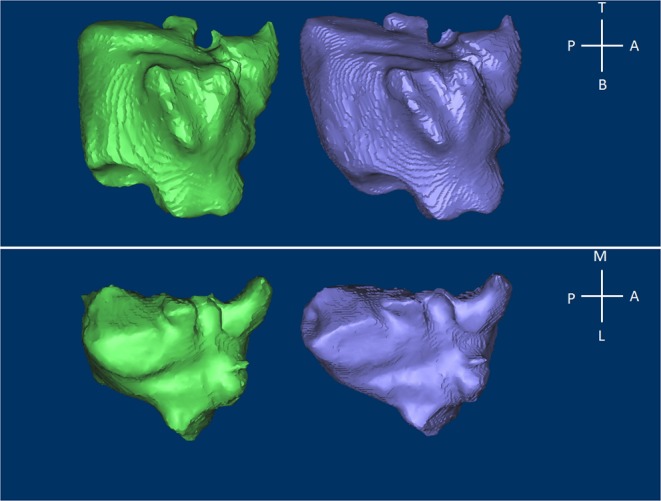
Three-dimensional morphological changes in the maxillary sinus between T0 (green) and T1 (purple). Above: lateral view; below: superior view. A: anterior; P: posterior; T: top; B: bottom; L: lateral; M: medial.

In the region of the maxillary dental arch between T0 and T1, the forward displacement of the P_6M_-point was significantly greater compared with the P-point (P < 0.001), whereas the opposite was found in downward displacement (P < 0.05) in UCLP and BCLP. The distance of A_1_-P_6M_⊥CR exhibited a mild decrease in UCLP (P > 0.05) and greater decrease in BCLP (P < 0.05), which were both mainly due to the significant decrease in A_1_-P_3M_⊥CR (P < 0.05) and relatively minor changes in P_3M_-P_6M_⊥CR (P > 0.05). In UCLP and BCLP, the width of anterior the dental arch was widened with a significant increase of P_3_W (P < 0.05), but the effect was not observed in the posterior dental arch of P_6_W (P > 0.05) (Fig. 2; Table 1). In the region of the maxillary tuberosity, the distance of P_6M_-P⊥CR exhibited a significant increase with an average of 4.32 ± 1.90 mm in UCLP (P < 0.001) and 5.34 ± 1.27 mm in BCLP (P < 0.001) (Fig. 2; Table 1). In addition, the 3D morphology of the maxillary sinus was altered and characterized by an elongated anteroposterior diameter and elevated anterior wall (Fig. 3). The maxillary sinus volume was also significantly increased both in UCLP (P < 0.001)(left side: 1920.73 ± 1345.61 mm^3^; right side: 2004.72 ± 1610.87 mm^3^) and BCLP (P < 0.05)(left side: 3346.42 ± 1799.01 mm^3^; right side: 3166.21 ± 2082.71 mm^3^) on either side when compared between T0 and T1 (Table 1).

### Contribution ratio of the advancement of the maxillary first molar

Between T0 and T1, the maxillary first molar showed a significant forward displacement with an average advancement of 16.71 ± 5.82 mm at the P_6M_-point in UCLP (P < 0.001) and 21.61 ± 5.95 mm in BCLP (P < 0.001). During this process, the contribution ratio of the advancement of P_6M_-P⊥CR and P⊥CR to P_6M_⊥CR were 26% and 74%, respectively, in UCLP and 25% and 75%, respectively, in BCLP (Table 1).

## Discussion

Midfacial hypoplasia in CLP patients results from a combination of intrinsic growth deficiency and iatrogenic factors. However, the latter exhibits greater interference effects on the growth and development of midfacial skeleton, whereas the former is still debated by multiple groups^20–22^. Of note, although the postsurgical scar tissue on the palate and lip restrained not only the growth modelling of circummaxillary sutures but also the midfacial skeleton itself, fortunately the growth potential of these structures stimulated by external distraction force was retained in growing CLP patients, and a series of maxillary protraction therapy and bone-borne TSDO have been developed based on this mechanism.

Numerous studies reported the maxillary growth response to various types of maxillary protraction therapy using 2-dimensional lateral cephalometric measurements, and similar skeletal changes were observed in the horizontal advancement of the A-point and the counterclockwise rotation of the occlusal plane despite differences between these treatment protocols^12–16^. However, in the zygomatic and infraorbital regions, contradictory results appeared due to the limitations of cephalometric 2-dimensional evaluation^23–25^. With the introduction of 3D image analysis, Heymann *et al*.^19^ and Yatabe *et al*.^20^ reported forward displacement of the zygomatic and infraorbital region in Class III patients and CLP patients, respectively, who underwent bone-anchored maxillary protraction with intermaxillary elastics to miniplates. Recently, our centre had also analysed the 3D midfacial skeletal changes after bone-borne TSDO therapy in growing CLP patients with severe midfacial hypoplasia, and the results revealed rotation advancement of the whole midfacial skeleton with progressively increased forward displacement from the top down along the midface segment and downward displacement in posterior part of the maxillary^17^. To date, these studies only presented the superficial skeletal changes after therapy, but the reason behind such a phenomenon remains unclear. Given that the success of therapy is highly dependent on the immature circummaxillary sutures and the midfacial skeleton itself, we conducted a 3D image analysis on the changes of these internal structures in the present study.

The circummaxillary sutures act as an interface for the displacement of adjacent bone through new bone formation under distraction forces. In the region of the cranial group, the results revealed forward and downward displacement at the pterygomaxillary, zygomaticotemporal and zygomaticofrontal suture in descending order with 20–30° anteroinferior distraction force vector to the occlusal plane at the lateral nasal wall. Accordingly, maximum new bone formation was observed in the pterygomaxillary suture followed by zygomaticotemporal and zygomaticofrontal suture. This order was mainly due to the differential stress values according to the distance from the stress centre and structural complexity of each suture, which was previously described in detail by 3D finite element analysis. Lee *et al*.^26^ reported maximum Von Mises stresses at the pterygomaxillary, zygomaticotemporal and zygomaticofrontal suture in descending order in both the lateral nasal wall and infrazygomatic area model in a Class III patient. Yang *et al*.^27^ revealed similar stress distributions at these sutures regardless of the anchorage methods and alveolar bone graft with CLP. Therefore, it might be concluded that this differential bone formation content with maximum in the pterygomaxillary suture mainly promoted the rotation advancement of the whole midfacial skeleton relative to the cranial base and caused the maximum forward displacement at the dentoalveolar level and the counterclockwise rotation of the occlusal plane. In the region of the midfacial group, the sutures were influenced by the anteroinferior pull of external distraction forces and the anterosuperior push from the cranial group. The combined effects results demonstrated that the internasal and nasomaxillary suture exhibited forward and upward displacement, whereas the zygomaticomaxillary suture exhibited significant major forward displacement. Together with the minor upward displacement of the A-point, these findings indicate that the maxillary basically maintained the forward displacement to minimize the counter-clockwise displacement under the resultant forces. These findings also indicate that the lateral nasal wall of the maxillary might be a proper site for bone anchorage, which was also supported by other authors^28^.

New bone deposition in the posterior border of the maxillary tuberosity could promote increased maxillary length. When interpreting the measurement results in the maxillary region, significantly increased advancement of the P_6M_-point was noted compared with the P-point. Further analysis of the contribution ratio values of the advancement of P_6M_-P⊥CR and P⊥CR to P_6M_⊥CR were 26% and 74%, respectively, in UCLP and 25% and 75%, respectively, in BCLP. These findings implied that approximately three-quarters of maxillary first molar advancement could be attributed to secondary displacement promoted by new bone formation in pterygomaxillary suture and that one-quarter could be attributed to primary displacement by the growth of the maxillary tuberosity. In addition, given the bone anchorage at the lateral nasal wall, the maximum stress in the paranasal area adjacent to the pyriform along with the orthodontic treatment in some patients resulted in significant alveolar bone remodelling around the canine associated with a widened anterior dental arch. The results of significant decrease of A_1_-P_3M_⊥CR in BCLP was mainly due to the separation of the premaxilla from the bilateral maxillary without alveolar bone graft.

In present study, the maxillary sinus, an important structure of the midface, was adaptively altered with regard to morphology and volume. Erdur *et al*.^29^ and Demirtas *et al*.^30^ reported that maxillary sinus volume was negatively affected in UCLP compared with the healthy control group. Hypoplasia of the maxillary caused hypoplastic maxillary sinus. Similarly, volume increases of maxillary sinus could promote the expansion of maxillary size. The findings indicate that the morphology of the maxillary sinus exhibited an elongated anteroposterior diameter associated with significant volume increases, suggesting that the growth of maxillary size mainly occurred with regard to length.

This study was the first attempt to explore the morphological changes of circummaxillary sutures, the maxillary tuberosity and the maxillary sinus in growing CLP patients with severe midfacial hypoplasia who underwent our bone-borne TSDO therapy. The main limitation of this study was the absence of an untreated control group. Therefore, it is difficult to evaluate what the observed changes in these structures are the result of the TSDO therapy versus normal growth. In addition, the effect of TSDO therapy can be affected by various patient- and treatment-related factors, including patient’s age, cleft type, cleft repair methods, amount of postsurgical scar tissue, distraction protocol and orthodontic treatment, and the relationship between them are still unclear. In this study, there were 5 patients who had an alveolar bone graft before TSDO therapy and 15 patients who had underwent orthodontic treatment during the distraction after evaluation by the team orthodontist. These treatments are very likely to affect the degree of skeletal changes. Although the findings of this study should be generalized cautiously due to the retrospective design and relatively small sample size, it still provided some useful information regarding the mechanism of midface growth under the action of external distraction forces. Further large-sample controlled studies with respect to various patient- and treatment-related factors are needed to improve our knowledge of such techniques.

In conclusion, according to the results of our preliminary imaging study, midface growth with bone-borne TSDO therapy in growing CLP patients depends on both the secondary displacement promoted by sutural bone formation mainly in the pterygomaxillary suture and the primary displacement by growth of maxillary tuberosity and maxillary sinus volume.

## Methods

### Study design and patients

The initial subjects were 73 growing CLP patients who underwent bone-borne TSDO therapy to correct midfacial hypoplasia at the Department of Cleft Lip and Palate, Plastic Surgery Hospital, Chinese Academy of Medical Science and Peking Union Medical College from January 2005 to December 2017. The inclusion criteria were as follows: growing nonsyndromic CLP patients with midfacial hypoplasia identified by clinical profile evaluation associated with skeletal Class III malocclusion; the same bone-borne TSDO therapy protocol undertaken by the same surgeon; complete skull CT images obtained preoperatively (T0) and immediately the day after the devices were removed (T1). Exclusion criteria were patients with incomplete or poor-quality data records and treatment-related complications.

**Figure 4:**
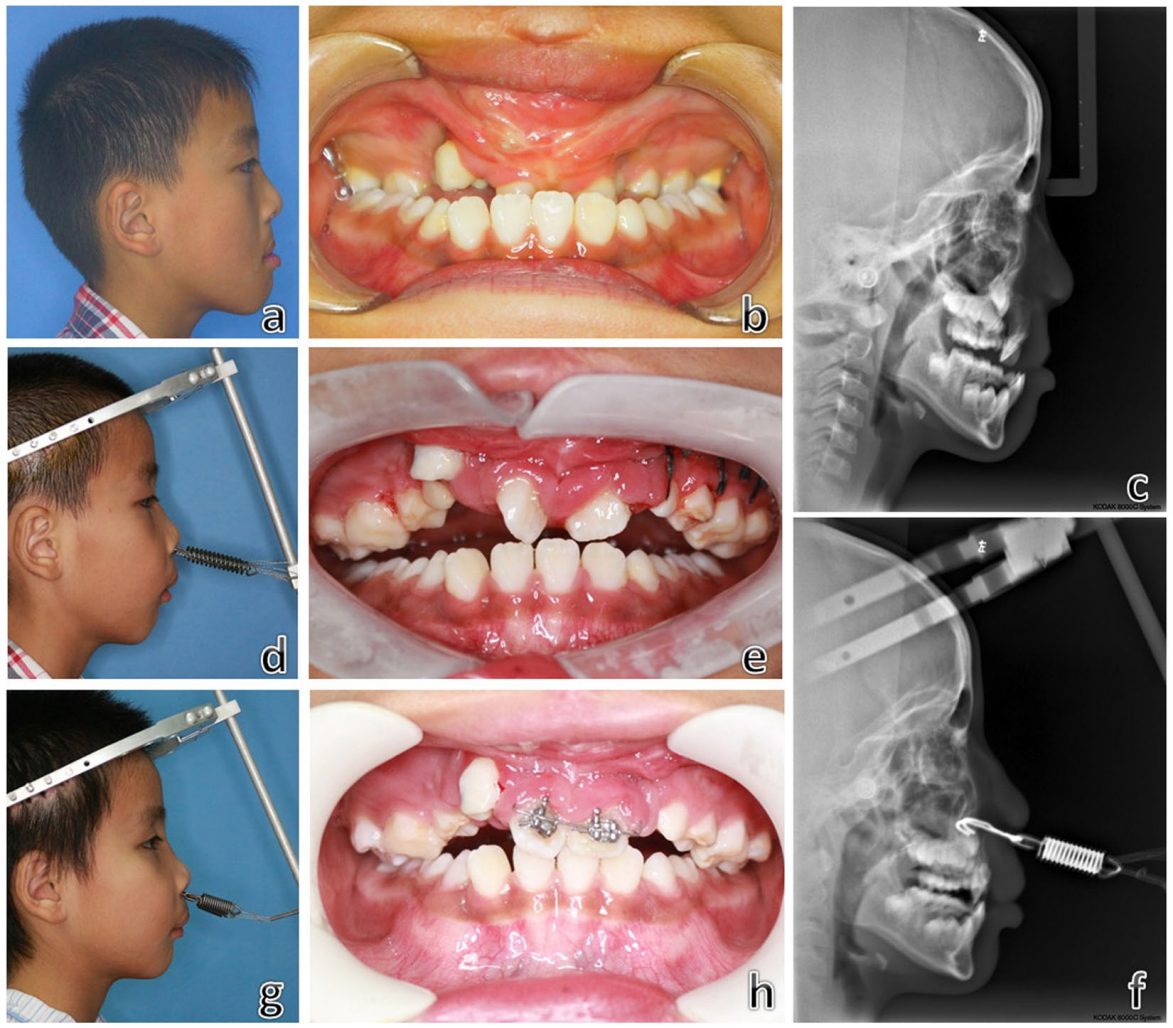
Views and cephalograms of a 10-year-old bilateral cleft lip and palate patient with midfacial hypoplasia. Preoperatively (**a**–**c**); during the distraction (**d**–**f**) and towards the end of distraction (**g**,**h**).

**Figure 5:**
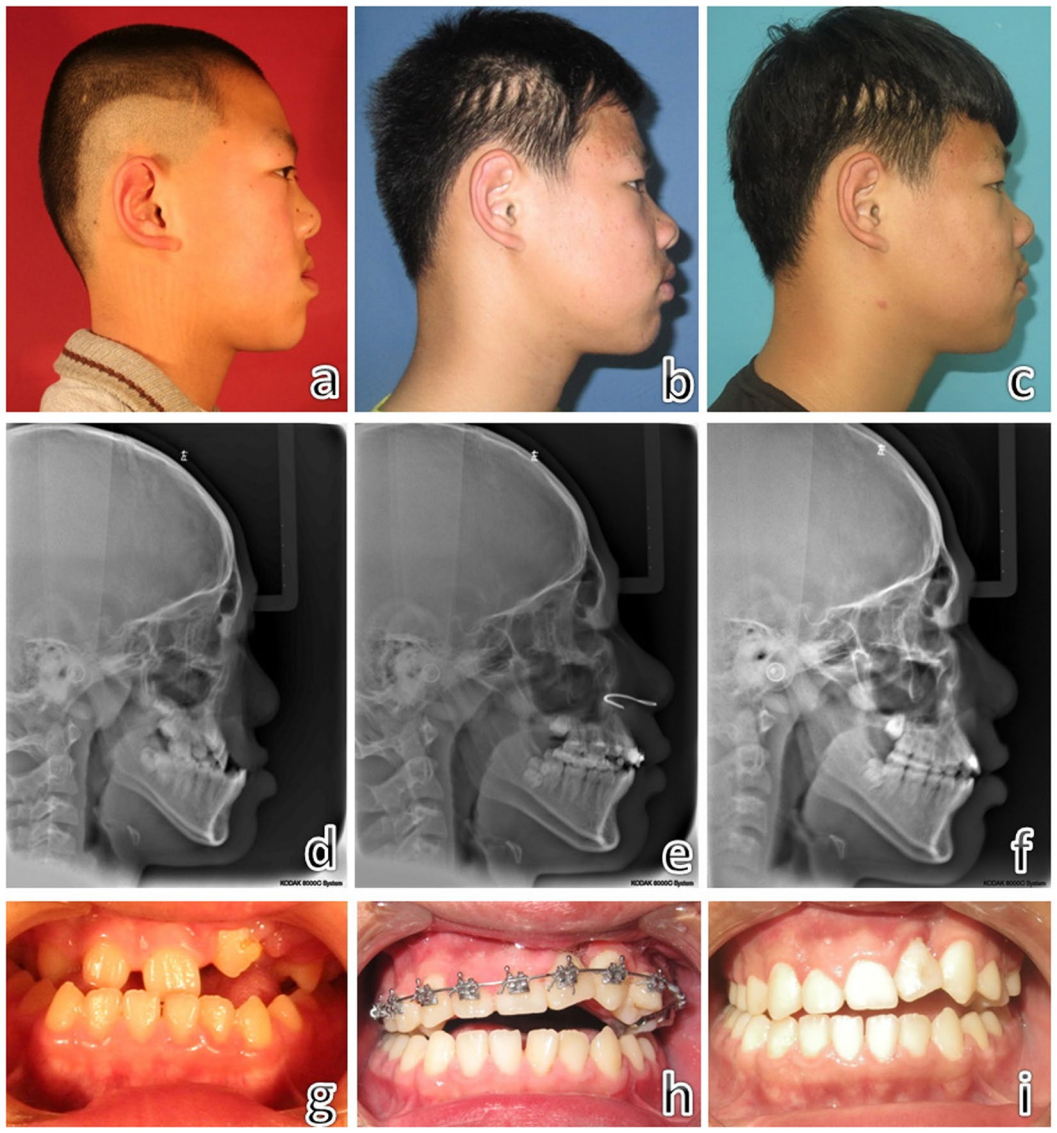
Views and cephalograms of an 11-year-old unilateral cleft lip and palate patient with midfacial hypoplasia. Preoperatively (**a**,**d**,**g**); 2 years post-distraction (**b**,**e**,**h**) and 3 years post-distraction (**c**,**f**,**i**).

The final subjects included 26 CLP patients (20 UCLP: left side 13, right side 7; 6 BCLP; 23 males and 3 females). The average age at the time of distraction was 11.5 ± 2.1 years (range, 8 to 15 years). There were 5 patients (5 UCLP) who previously had an alveolar bone graft from the iliac crest and 15 patients (12 UCLP and 3 BCLP) who underwent orthodontic treatment accompanied with the distraction. The average distraction time was 40.0 ± 11.5 days (range, 24 to 64 days) and the average unilateral maximum traction force was 4.45 ± 0.87 kg (range, 3 to 6 kg). Typical cases are shown in Figs 4 and 5.

This retrospective study was approved by the Institutional Review Board of Plastic Surgery Hospital, Chinese Academy of Medical Science and Peking Union Medical College, and the study was performed according to the principles of the Declaration of Helsinki. Informed consents were obtained from the parents or legal guardians of patients for both study participation and publication of identifying information/images in an online open-access publication.

### Surgical technique and distraction protocol

Under general anaesthesia, a bone hole was drilled on each side approximately 1 cm outside of the lateral pyriform rim and 5 mm above the apices of the maxillary teeth through the lateral nasal wall. Two independent traction hooks (GEE Co., Beijing, China) were introduced through the hole with the head end around the canine pillars as bone anchorage and the caudal ends extending out from the nostril base. Then the cranial frame of rigid external distractor (RED, Cibei Medical Treatment appliance Co., Ningbo, China) was installed and connected to the hooks via a nickel-titanium shape memory alloy spring (GEE Co., Beijing, China) which could generate a continuous and stable force of approximately 250 g/mm within a certain range of deformation. The initial direction of distraction force was adjusted 20–30°anteroinferiorly to occlusal plane.

Without a latency period, the distraction commenced immediately after the operation with an initial force of 750 g on each side and was maintained 3 to 5 days for patients to adapt. Then the traction force was gradually increased by adjusting the length of spring, with a variable rate of 1 to 2 mm every 2 to 3days mainly based on the patient’s adaptation and age. The maxillary moved forward with the traction force slowly increasing to maximum during the distraction phase until the required advancement was obtained. The adequate positive overjet with a moderate overcorrection and facial contour were used as the clinical guide to determine the end of the distraction. The system was then left in place for a consolidation period of 1–3 months with gradually decreased traction force. After removal of the device, a removable orthodontic facemask with elastic traction were instructed to use at night for those patients whose normal occlusal relationship could not be established in time after evaluation by the team orthodontist.

### CT image analysis

The skull CT images were obtained at T0 and T1 using the following protocol: exposure conditions, 120 Kv and 240 mA; slice thickness, 0.5 mm; image matrix size, 512 × 512; pixel size, 0.35 mm (Aquilion 64;Toshiba,Tokyo, Japan). CT data were stored in the Digital Imaging and Communications in Medicine file format (DICOM) and further analysed using Mimics 10.01 software (Materialise, Leuven, Belgium). This process involved the following steps:

1. The original slice images were imported into the software to create 3D models of skeleton and maxillary sinus by thresholding and then exported in binary STL files.
2. Using the STL global registration function, the T0 and T1 3D skeleton models were superimposed automatically with a minimal point distance filter setting at 1 mm. Visualization and assessment of treatment changes was performed using the superimposition 3D images.
3. To establish the standard orientation of the craniofacial structures, 3D reference planes were initially created by the anatomical landmarks of T0 for quantitative measurement of both T0 and T1 3D models (Table 2).

**Table 2:** Landmarks, 3D reference planes and measurements used in the present study.

Landmark	Definition
Point	
S (Sella)	Centre of the pituitary fossa
N (Nasion)	Intersection of internasal suture and frontonasal suture
Po (Porion)	Most superior point on roof of external auditory meatus
Or (Orbitale)	Most inferior point along the infraorbital rim
Ba (Basion)	Midpoint on the forward border of foramen magnum
A (Subspinale A-point)	Most posterior point on profile of maxillary between anterior nasal spine and alveolar crest
A_1_	Most inferior and anterior point on alveolar crest of the maxillary
P_3_	Midpoint of palatally gingival margin of maxillary canine
P_6_	Midpoint of palatally gingival margin of maxillary first molar
Ptm	Most inferior point on profile of pterygomaxillary suture
P_3M_	Midpoint of the line between bilateral P_3_
P_6M_	Midpoint of the line between bilateral P_6_
P	Midpoint of the line between bilateral Ptm
3D Reference Plane	
Frankfurt horizontal plane (FH plane)	Plane consisting of both sides of Po and Or of non-cleft side (UCLP)or left side (BCLP)
Horizontal reference plane (HR plane)	Parallel to FH plane, passing through N
Midsagittal reference plane (MSR plane)	Perpendicular to HR plane, passing through Ba and S
Coronal reference Plane (CR plane)	Perpendicular to HR and MSR plane, passing through S
Measurement	
P_3_W (mm)	Width of anterior dental arch between bilateral P_3_
P_6_W (mm)	Width of posterior dental arch between bilateral P_6_
A_1_-P_3M_⊥CR (mm)	Distance from A_1_ to P_3M_⊥CR plane
A_1_-P_6M_⊥CR (mm)	Distance from A_1_ to P_6M_⊥CR plane
P_3M_-P_6M_⊥CR (mm)	Distance from P_3M_ to P_6M_⊥CR plane
P_6M_-P⊥CR (mm)	Distance from P_6M_ to P⊥CR plane
P_6M_-P⊥CR/P_6M_⊥CR	Contribution ratio of the advancement of P_6M_-P⊥CR to P_6M_⊥CR between T0 and T1
P⊥CR/P_6M_⊥CR	Contribution ratio of the advancement of P⊥CR to P_6M_⊥CR between T0 and T1
V_MS_ (mm^3^)	Volume of maxillary sinus
4. The anatomical landmarks for 3D measurements, as described and summarized in Table 2, were first plotted on the surface of the 3D model and their positions were calibrated in the multiple planar reconstruction views. The distance of each landmark to the 3D reference planes and further linear and volumetric parameters were automatically measured and calculated. All data were then saved in a comma-separated value (.csv) file and transferred to the computer for statistical analysis.

### Circummaxillary sutures grouping

The circummaxillary sutures were divided into two groups according their anatomical position: (a) Cranial group: sutures connecting the midfacial skeleton to cranial base structures including pterygomaxillary zygomaticotemporal, zygomaticofrontal, frontomaxillary and frontonasal suture. (b) Midfacial group: sutures connecting each segment of the midface into a whole, including internasal, nasomaxillary and zygomaticomaxillary suture.

### Statistical analysis

Statistical analyses were performed using the Statistical Package for Social Science software version 17.0 (SPSS Inc., Chicago, Ill). To evaluate the reliability and reproducibility of the CT image analysis results, the identification of landmarks and measurements of parameters were repeated after a 2-week interval in 10 randomly selected images. The intraclass correlation coefficient test (ICC) ranged from 0.92 to 0.98 (intraobserver) and 0.86 to 0.94 (interobserver), indicating a high level of reliability and reproducibility. Comparisons between T0 and T1 were conducted using the paired-samples *t*-test. A level of P < 0.05 was considered statistically significant.

## Data Availability

The datasets generated or analysed during the current study are available in https://pan.baidu.com/s/1XitvDrQwLIlMOhnSCKGu5A.
